# Letters and Opinions by Early-career Writers

**DOI:** 10.31662/jmaj.2025-0038

**Published:** 2025-03-28

**Authors:** Soichiro Saeki

**Affiliations:** 1 Emergency Medicine and Critical Care, Center Hospital of the National Center for Global Health and Medicine, Tokyo, Japan; 2 Division of Public Health, Department of Social Medicine, Graduate School of Medicine, Osaka University, Osaka, Japan

**Keywords:** academic writing, medical education, postgraduate education, undergraduate education, manuscript

I thank Prof. Matsubara ^(1)^ for the comments on my previous manuscript ^(2)^, recommending that less-experienced writers focus on writing “Opinion” letters and base their content on their unique “younger sensitivity.” I endorse these recommendations. The checklist in the previous manuscript was created so that potential authors can have a reference to refer to when writing Opinions, not only Agreement/Disagreement letters ^(1)^.

Opinions provide platforms for less-experienced (early-career) medical professionals to share their perspectives, a vital role that Opinions fulfill. Early-career medical professionals, positioned at the forefront of clinical practice, contribute fresh insights that hold significant value for the medical community. In this context, young medical professionals should not only aspire to write opinion pieces but also consider contributing to Agreement/Disagreement letters. The clinical experience gained through direct patient care is invaluable, and the ideas born from such encounters are worth scholarly discussion.

In contrast, when an Agreement/Disagreement letter is rejected, it is often difficult to revive. This challenge similarly applies to opinion pieces, as identifying an appropriate alternative journal for resubmission can be difficult. Hence, journal editors should provide comments or feedback when rejecting submissions of Letters or Opinions from early-career researchers. Such feedback would allow the authors to gain valuable learning experiences and inspire their motivation for future scholarly endeavors ^(3)^. To achieve this, it is essential to recognize letters as formal academic achievements. As viewing Letters as inferior to original research articles regarding academic merit is customary, creating a culture where letters are acknowledged as meaningful contributions would encourage researchers to engage more actively in such submissions. If a letter is unfortunately rejected, there are two possible paths forward. The first is to broaden the scope of the discussion and refine the content into an Opinion. The second is to utilize platforms like PubPeer (https://pubpeer.com/) to share the content and engage in academic discussions with other researchers.

I am deeply grateful for my growth experience writing Letters and Opinion pieces. As a medical student, I wrote my first Letter ^(4)^ and Opinion ^(5)^ with my colleagues, and it was only after becoming a resident physician that my first original research article was accepted. Undoubtedly, the experience of writing these earlier pieces contributed significantly to my ability to complete an original research article. I sincerely hope that the Japanese medical community will continue to provide an environment where motivated young researchers are warmly supported and can flourish through opportunities for academic growth.

## Article Information

### Conflicts of Interest

None

### Acknowledgement

The author thanks his colleagues for their helpful discussions on this topic. The author acknowledges the use of Grammarly (Grammarly Inc, San Fransico, USA) for primary language editing. The views expressed in this manuscript are those of the author and do not necessarily represent the author’s institutions.

### Author Contributions

The author is solely responsible for the manuscript content. Artificial intelligence technology was used for language editing, and such content was reviewed by the author. The author’s institution played no role in the conceptualization of this manuscript.

### Approval by Institutional Review Board (IRB)

Not applicable.

## References

[1] Matsubara S. Less-experienced writers’ writing letters: some concerns and recommendations. JMA J. 2025;8(2):667-668. 10.31662/jmaj.2025-0015 PMC12095135 40416023

[2] Saeki S. Presenting new ideas and opinions in academic journals. JMA J. 2025;8(1):310-1. 39926093 10.31662/jmaj.2024-0339 PMC11799405

[3] Saeki S. Encouraging students and trainees to write. JMA J. 2023;6(1):73-5. 36793533 10.31662/jmaj.2022-0134 PMC9908373

[4] Yamasaki L, Saeki S, Kido H, et al. Medical students in the midst of COVID-19 -Report of the joint student symposium in the Joint Congress of Global Health 2020 in Osaka. J Int Health. 2020;35(4):265-6.

[5] Saeki S, Shimato M. Mental health support for the current and future medical professionals during pandemics. JMA J. 2021;4(3):281-3. 34414324 10.31662/jmaj.2021-0039 PMC8355721

